# SAGE2Splice: Unmapped SAGE Tags Reveal Novel Splice Junctions

**DOI:** 10.1371/journal.pcbi.0020034

**Published:** 2006-04-28

**Authors:** Byron Yu-Lin Kuo, Ying Chen, Slavita Bohacec, Öjvind Johansson, Wyeth W Wasserman, Elizabeth M Simpson

**Affiliations:** 1 Genetics Graduate Program, University of British Columbia, Vancouver, British Columbia, Canada; 2 Centre for Molecular Medicine and Therapeutics, Child and Family Research Institute, Department of Medical Genetics, University of British Columbia, Vancouver, British Columbia, Canada; 3 Stockholm Bioinformatics Center, Kunliga Tekniska Högskolan, Albanova, Stockholm, Sweden; National Center for Genome Resources, United States of America

## Abstract

Serial analysis of gene expression (SAGE) not only is a method for profiling the global expression of genes, but also offers the opportunity for the discovery of novel transcripts. SAGE tags are mapped to known transcripts to determine the gene of origin. Tags that map neither to a known transcript nor to the genome were hypothesized to span a splice junction, for which the exon combination or exon(s) are unknown. To test this hypothesis, we have developed an algorithm, SAGE2Splice, to efficiently map SAGE tags to potential splice junctions in a genome. The algorithm consists of three search levels. A scoring scheme was designed based on position weight matrices to assess the quality of candidates. Using optimized parameters for SAGE2Splice analysis and two sets of SAGE data, candidate junctions were discovered for 5%–6% of unmapped tags. Candidates were classified into three categories, reflecting the previous annotations of the putative splice junctions. Analysis of *predicted tags* extracted from EST sequences demonstrated that candidate junctions having the splice junction located closer to the center of the tags are more reliable. Nine of these 12 candidates were validated by RT-PCR and sequencing, and among these, four revealed previously uncharacterized exons. Thus, SAGE2Splice provides a new functionality for the identification of novel transcripts and exons. SAGE2Splice is available online at http://www.cisreg.ca.

## Synopsis

Serial analysis of gene expression (SAGE) analysis is used to profile the RNA transcripts present in a cell or tissue sample. In SAGE experiments, short portions of transcripts are sequenced in proportion to their abundance. These sequence tags must be mapped back to sequence databases to determine from which gene they were derived. Although the present genome annotation efforts have greatly facilitated this mapping process, a significant fraction of tags remain unassigned. The authors describe a computational algorithm, SAGE2Splice, that effectively and efficiently maps a subset of these unmapped tags to candidate splice junctions (the edges of two exons). In two test cases, 7%–8% of analyzed tags matched potential splice junctions. Based on the availability of RNA, sufficient information to design polymerase chain reaction (PCR) primers, and the confidence score associated with the predictions, 12 candidate splice junctions were selected for experimental tests. Nine of the tested predictions were validated by PCR and sequencing, confirming the capacity of the SAGE2Splice method to reveal previously unknown exons. Using recommended high specificity parameters, 5%–6% of high-quality unmapped SAGE tags were found to map to candidate splice junctions. An Internet interface to the SAGE2Splice system is described at http://www.cisreg.ca.

## Introduction

The complexity of the transcriptome is significantly greater than that of the genome due to alternative splicing. It is estimated that between 35%–65% of human genes are alternatively spliced [1,2]. The *slo* gene, for example, is estimated to produce more than 500 distinct transcripts, which regulate various responses of the hair cells of the inner ear to sound [3]. Identification of the transcripts present within a cell can provide insights into the regulatory processes that control the cell-specific interpretation of the genome [4].

Serial analysis of gene expression (SAGE), in which a representative tag (14 to 26 base pairs [bp]) is excised from each transcript, is a powerful and efficient technology for high-throughput qualitative and quantitative profiling of global transcript expression patterns [5]. SAGE quantitatively measures transcript levels, providing the absolute number of each transcript-specific tag within a library of all tags. That no prior knowledge of the transcripts being studied is required makes SAGE advantageous over array-based methods for the discovery of novel transcripts [6–11].

An essential step in the analysis of SAGE data is the assignment of each tag to the transcript from which it was derived [10]. This process, termed *tag-to-gene mapping,* involves comparison of tag sequences to transcript databases. A commonly used technique is to compare SAGE tags to predicted tags (also known as *virtual tags*). Based on known transcript sequences, predicted tags are those expected to be generated by a SAGE protocol [12]. Often, the predicted tags closest to the 3′ end of transcripts are emphasized, because SAGE protocols impart a location bias. However, in a SAGE experiment, due to alternative splicing or incomplete enzyme digestion [13,14], tags can be excised from other positions. The choice of sequence databases impacts the quality of tag-to-gene mapping [10]. A highly curated and more complete transcriptome database not only facilitates mapping of more tags, but also increases confidence in the mappings. Many resources have been developed for mapping SAGE tags to genes, including SAGEmap from the National Center for Biotechnology Information (NCBI) [15], the National Institutes of Health Cancer Genome Anatomy Project's SAGE Genie [16], the Mouse SAGE Site [17], Identitag [12], and DiscoverySpace [18]. Despite these efforts, however, a major problem of tag-to-gene mapping exists as approximately one third of the tags are unmapped. Inability to map tags limits the information obtained in SAGE studies [6,7,10]. The identification of unmapped tags remains an active research topic in SAGE analysis.

Recent studies have attempted to map SAGE tags that did not match the known transcriptome. Chen et al. [19] studied 1,000 unmapped SAGE tags from publicly available libraries by generating longer cDNA fragments from SAGE tags for gene identification (GLGI), and concluded that 67% of the unmapped tags originated from novel transcripts. In an analysis of unmapped long SAGE tags (21 bp), Saha et al. [20] predicted 60% were from transcripts of novel genes and 40% were from unidentified internal exons of predicted genes. Gorski et al. [8] identified 225 cases of genes that previously had been unidentified by gene prediction programs. Each of these studies affirmed the capacity of SAGE profiling to facilitate identification of novel transcripts.

Tags that do not map to the transcriptome or to the genome may span adjacent exons of which one or both were previously unidentified [8]. We analyzed predicted tags derived from known transcripts and observed that between 2% to 6% of these tags span a splice junction. Thus, even tags that do not map to the genome are anticipated to be a resource for the discovery of novel transcripts. To test our hypothesis, we developed an algorithm, SAGE2Splice, for mapping tags to potential splice junctions in a genome. Applying this new method for tag-to-gene mapping, we demonstrated that 5%–6% of unmapped tags span candidate splice junctions.

## Results

### Some Predicted SAGE Tags Span a Splice Junction

**Figure 1:**
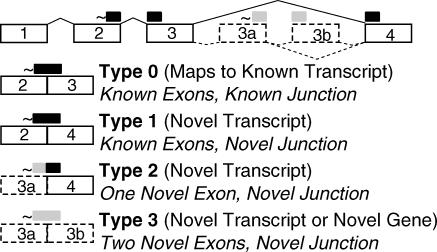
Tags That Span a Splice Junction May Reveal Novel Genes or Novel Transcripts This schematic demonstrates four known exons (1, 2, 3, and 4, boxes in solid lines). The 3′-most NlaIII enzyme restriction site (represented as ~) lies near the 3′ edge of exon 2 and a known predicted SAGE tag (long black bar) spans exons 2 and 3 (Type 0 tag). Predicted exons (boxes in dashed line) 3a and 3b are examples of exons predicted by SAGE2Splice. Three other types of tags (Types 1 to 3) have been defined as potential candidates in SAGE2Splice predictions. Tag portions arising from known exons (short black bar), whereas tag portions arising from novel exons (short gray bar). Solid lines connecting exons indicate known combinations, whereas dashed lines indicate unknown combinations.

We defined four distinct types of spliced tags, tags that span a splice junction (Figure 1). A Type 0 tag matches portions of two exons at a known splice junction. Type 0 tags were identified by mapping to known transcripts. A Type 1 tag also spans two known exons, but the junction is not present in the transcriptome databases. A Type 2 tag spans a previously known exon and a previously unknown exon. Both Type 1 and Type 2 tags indicate a novel transcript of a previously characterized gene. A Type 3 tag spans two previously unknown exons and indicates either two novel exons of a characterized gene, or two exons of a novel gene.

To determine the portion of predicted tags that span splice junctions of known transcripts, we studied the NCBI Reference Sequences (RefSeq). From 17,848 sequences studied, 198,419 predicted tags were extracted based on the identification of all NlaIII restriction sites. A total of 193 RefSeq sequences (approximately 1.08%) did not contain a NlaIII restriction site and thus were unable to give rise to a SAGE tag. Among the predicted tags, 12,297 (6.2%) overlapped a splice junction (Type 0). In addition, 14 predicted tags traversed two splice junctions (Table 1). These were due to very small exons [21], between 1 bp and 4 bp in length. Since the SAGE technique excises tags from the NlaIII restriction site closest to the 3′ end of transcripts, from the RefSeq sequences, 17,655 predicted tags were extracted from the 3′-most position and investigated. Among these predicted tags, only 292 (1.6%) were Type 0. The different Type 0 frequencies between the all-position set and the 3′-most set reflects that exons are generally longer at the 3′ end of a transcript [21]. In the analyzed RefSeq sequences, the average length of all exons was 262 bp, whereas the average for all 3′-most exons was 1,068 bp. Hence, at the 3′-most position, the probability of finding a splice junction within a tag is lower than that from the set of all NlaIII positions.

**Table 1:**
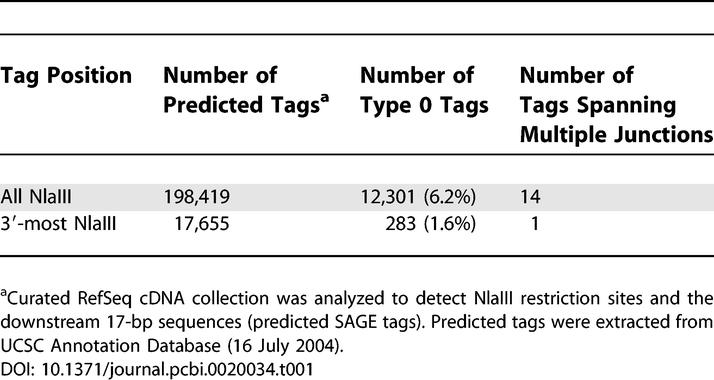
A Total of 6.2% of Predicted Tags from All NlaIII Restriction Sites and 1.6% from 3′-Most Sites Were Found to Span a Known Splice Junction (Type 0 Tags)

### Intron Properties

**Figure 2:**
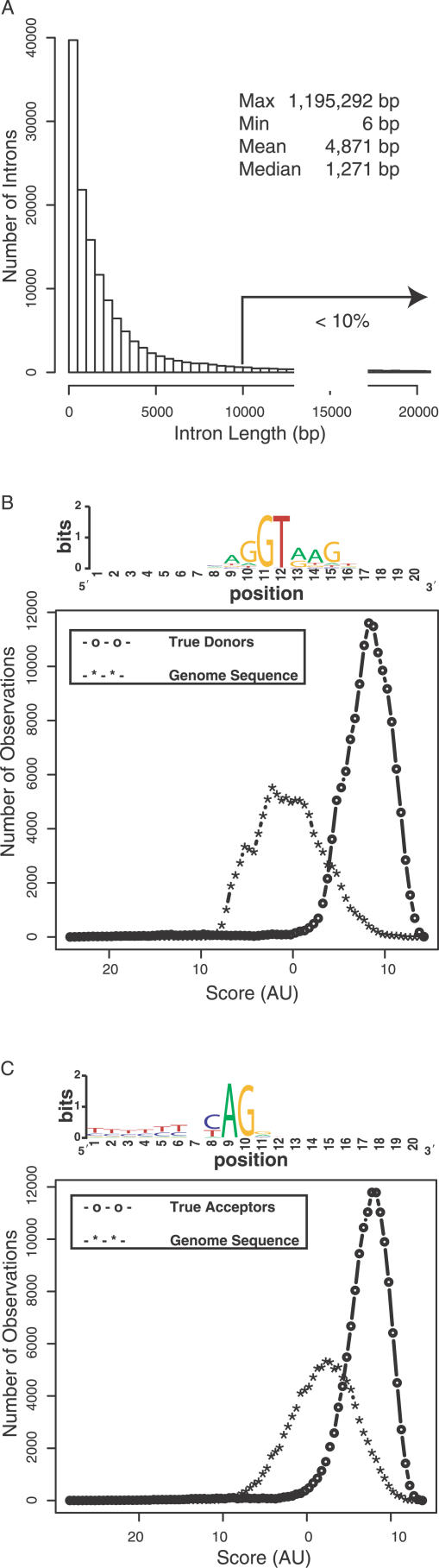
Length and Boundary Nucleotides of Introns Are Important Properties for Detecting a Splice Junction (A) Fewer than 10% of introns in RefGene annotation were greater than 10,000 bp in length. PWMs for splice junctions with respect to true donors (B) and true acceptors (C) were applied to true splice junctions defined by RefGene annotations and to randomly selected genome sequences containing the canonical dinucleotide pair at the appropriate position. The scores, which were computed based on the profile model, for donors and acceptors were plotted and showed that true splice junctions acquired high scores. The information content and the relative frequency of nucleotides at each position are measured in bits (vertical axis of the sequence logo diagrams) to indicate the strength of signals. Two bits of information are required to determine the content of a DNA sequence. AU, arbitrary units.

In our development of SAGE2Splice, an important search criterion was to determine the maximum length the algorithm should allow for candidate introns. Previous studies have shown that, although a typical intron is 40–125 bp in length, the average length is approximately 1,000 bp because the sizes of introns vary over a very wide range [21,22]. In our studies of the RefGene annotations, we confirmed that within the known splice junctions, introns vary from 6 to 1,195,292 bp in length, with a median of 1,271 bp (Figure 2). Ninety percent of introns were smaller than 10,000 bp and 95% were smaller than 20,000 bp. We incorporated 10,000 bp as the default for maximum intron size in the search for candidate splice junctions.

To gain a more detailed understanding of the sequence patterns of splice junctions, we examined 10 bp flanking each side of the donor junctions and 10 bp flanking each side of the acceptor junctions. For each junction type, we constructed a matrix representing the frequency of each nucleotide at each position. Position weight matrices (PWMs) were constructed by converting the frequencies into scores relative to the expected frequency of a randomly selected nucleotide (see Materials and Methods). By using these scoring matrices, we generated genuine score distributions for true splice junctions in RefSeq and empirical score distributions for randomly selected sequences from the genome. By superimposing the genuine distribution on the empirical distribution, it was shown that genuine splice junctions typically had high scores and were located on the far-right end of the empirical curve (Figure 2). Hence, we incorporated these properties into our SAGE2Splice algorithm for ranking and determining the likelihood of candidates.

### The SAGE2Splice Algorithm

#### Pre-processing the input SAGE tags.

**Figure 3:**
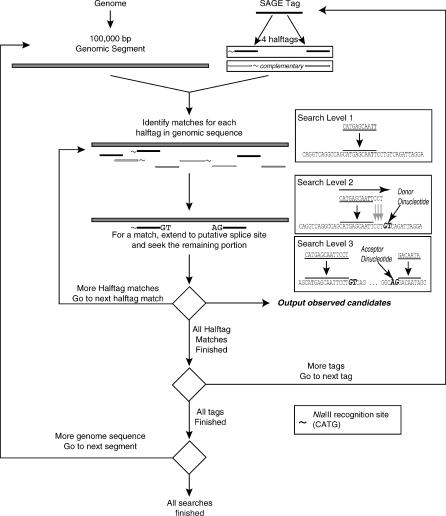
SAGE2Splice Algorithm Searches the Genome for Novel Splice Junctions By splitting each tag into two halftags and making complementary copies, the algorithm searches for candidate splice junctions against continuous segments of the genome in three progressive steps. After each level, if the matching criteria are fulfilled, the algorithm goes on to the next level. If criteria are not fulfilled, the algorithm analyzes the next tag. Once all tags have been analyzed, the next genomic segment is read, and the algorithm returns to the first level.

In a 21-bp SAGE tag, if a splice junction exists within the sequence, one of the two portions is no shorter than 11 bp in length. Each 21-bp tag is therefore split into two equal portions of 11 bp (overlapping by one bp), which are used as search strings simultaneously. We term these equal-sized portions as the *halftags*. Prior to a search, complementary sequences for the halftags were constructed because genes can be located on either strand of the genome. The program reads the sequences of each chromosome one segment of 100,000 bp at a time. To perform a complete search, the algorithm holds three such segments in memory at any one time: the previous segment, the current segment, and the next segment. Searching for a candidate splice junction in SAGE2Splice consists of three progressive levels (Figure 3). At each level, only if the defined matching criteria are fulfilled will the algorithm proceed to the next level. Otherwise, the algorithm imports a new segment of the genome into memory, and the search starts over from the first level.

#### Search Level 1: Matching halftags.

In Search Level 1, SAGE2Splice searches each halftag against the current segment by using the pattern-matching function built into the Perl programming language (version 5.6). Positions of all matches are stored as a tab-delimited string. A complementary halftag match, indicating a position on the complementary strand, is stored as a negative position. If at least one halftag match is found, the algorithm proceeds to Search Level 2. Otherwise, the next segment of the chromosome is imported, and the search for candidate splice junctions returns to Search Level 1.

#### Search Level 2: Extending halftags.

SAGE2Splice searches for one boundary of a potential candidate intron before searching for the other boundary. During Search Level 2, SAGE2Splice attempts to find, for each halftag match, one of the edges of a potential intron. From Search Level 1, a 5′ halftag match to the genomic segment indicates a search of a potential donor intron-exon boundary in Search Level 2. Conversely, a 3′ halftag match suggests a search for the acceptor boundary. Hence, in the second level, the SAGE2Splice algorithm extends the first level halftag match, base by base against the original tag. At every base extension, depending on whether or not the halftag match is 5′ or 3′, the respective intron boundary dinucleotide is added and matched to the genome segment. As a result, all potential candidates for one edge of an intron are discovered for every halftag match. For the 5′ halftag match, the extension is toward the 3′ end and the donor dinucleotide is GT, whereas for the 3′ halftag match, the extension is toward the 5′ end and the acceptor dinucleotide is AG. A match of the complementary halftags indicates a potential candidate on the complementary strand of the genome sequence and, thus, the base extension direction is opposite that of the sense strand. If a potential intron–exon boundary is found, the algorithm continues to Search Level 3. Otherwise, SAGE2Splice reads the next genomic segment and returns to Search Level 1.

#### Search Level 3: Searching remaining portions.

In Search Level 3, the remaining tag portion for the corresponding candidate splice junction is sought within 10,000 bp, or a maximum distance set by the user. If the preceding level found a candidate donor junction, the search looks for candidate acceptor junctions with the conserved dinucleotide, AG, toward the 3′ direction, in accord with the definition of splice junctions [22]. If, on the other hand, the previous search returned a candidate acceptor junction, the search for candidate donors is toward the 5′ direction and the conserved dinucleotide is GT. Searches for the remaining tag portions for the complementary halftag are in the opposite direction. When a candidate splice junction is returned, the algorithm proceeds to scoring and ranking the candidate. Because a match in Search Level 1 could be close to the edges of the current genomic segment, having the previous and the next segments in memory allows for potential matches located beyond the current segment. If, however, Search Level 3 does not return a candidate splice junction, the search returns to Search Level 1 to start on a new segment of the chromosome.

### Scoring Candidate Splice Junctions

Once a candidate is discovered and returned by Search Level 3, for both the donor and the acceptor, 10 bp flanking each side of the boundary are extracted and evaluated using the respective PWM. Probability values (*p-*values) are generated by determining the position of the observed scores within the empirical score distributions. For a tag that matches multiple candidates, SAGE2Splice ranks the candidates according to the composite *p-*value. After this process, SAGE2Splice returns for each candidate the following information to the user: the chromosome number; the two tag portions with their positions, scores, and *p-*values; the composite *p-*value; and the predicted intron length.

### Efficiency Tuning of SAGE2Splice

**Figure 4:**
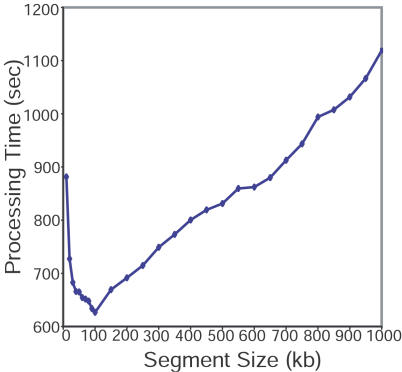
SAGE2Splice Was Optimized for Processing Time by Using Different Genomic Segment Lengths (Ranging from 10 kb to 1,000 kb) For SAGE2Splice performance, 100 kb was determined as the optimal size.

Five parameters affect the performance of SAGE2Splice, including the number of SAGE tags in the search, the length of SAGE tags, the cutoffs for *p-*values, the cutoff for maximum intron length, and the length of genomic segment in memory. Other than the length of genomic segment in memory, all factors depend on either the input SAGE tags or user-specified parameters. We investigated the use of genomic segments of different lengths to fine-tune SAGE2Splice for best performance (Figure 4). The total execution time of SAGE2Splice decreased until it reached a segment size of 100,000 bp, and linearly increased thereafter.

### Sensitivity and Specificity

**Figure 5:**
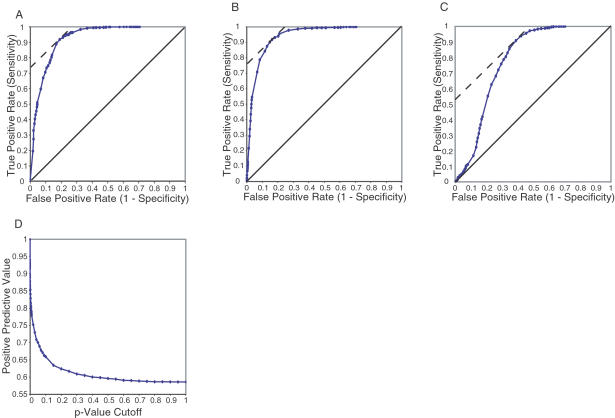
SAGE2Splice Achieves High Sensitivity but Relatively Low Specificity (A) The area under the ROC curve is 0.9232, indicating a candidate found by SAGE2Splice was much better than expected by random chance. Conversely, to achieve high specificity, the sensitivity (true positive rate) was significantly compromised. The tangent point of the dashed line is the optimal point when the costs of misclassifying positive and negative candidates are equal. This point corresponds to a *p-*value cutoff of 0.0025. (B) Analysis of the ROC curve for the donor splice junctions indicates a cutoff *p-*value of 0.06 as the optimal point. (C) For the acceptor splice junctions, the optimal cutoff *p-*value is determined to be 0.15. (D) The positive predictive value indicates that a high probability (greater than 0.9) of correct predictions requires a restrictive *p-*value (less than 0.0001).

To test the accuracy of SAGE2Splice and determine the optimal parameter settings, we investigated the sensitivity and the specificity for various *p-*value cutoffs, ranging from 0.00001 to 1. The receiver operating characteristic (ROC) curve demonstrates a tradeoff between sensitivity and specificity (Figure 5). As we varied the overall *p-*value cutoffs, it was observed that when a specificity of close to 95% was achieved, sensitivity dropped to 55%. The ROC curve shows that, although SAGE2Splice can achieve high sensitivity, specificity suffers dramatically at such settings. Moreover, the positive predictive value, which indicates the proportion of the candidates that are true positives, decreases as the *p-*value cutoffs increase (Figure 5). Such results correspond to previous studies [23,24] that showed that true splice junctions acquire high profile scores in the evaluation scheme and, thus, candidates with lower *p-*values are more likely to be true. In the ROC curve, the point with the minimum number of misclassified candidates (defined by a tangent line for which the slope equals 1) occurs when the composite *p-*value cutoff is approximately 0.0025, leading to a sensitivity (true positive rate) of 0.9 and a specificity of 0.82 (false positive rate = 0.18) (Figure 5). Similarly, separate analyses of the donor junction and the acceptor junction revealed the optimal cutoffs to be 0.06 and 0.15, respectively.

### Edge Length and Rank Analysis

**Figure 6:**
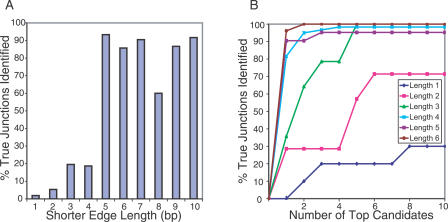
The Probability of Finding the True Splice Junction Is Lower if the Splice Junction Is Located Closer to the Edge of a Tag By using the unmapped tags in the Mouse Atlas Project that map to spliced tags predicted from EST transcripts, the percentage of true splice junctions found was analyzed for each short edge length. (A) By using high specificity parameters (cutoffs of 0.06, 0.15, and 0.25 for donor, acceptor, and composite *p-*values, respectively), 93% of the true splice junctions were found when the shorter edge is greater than or equal to 5 bp in length. (B) With no *p-*value cutoffs, 90% of the true splice junctions were found with the top-ranked *p-*value when the shorter edge is 5 bp in length.

To analyze the relationship between search accuracy and the position of a splice junction within a splice tag, we obtained expressed sequence tag (EST) transcript annotations from the University of California Santa Cruz, (UCSC) Genome Browser (http://genome.ucsc.edu) and extracted Type 0–predicted tags that had GT and AG for the donor and acceptor boundary dinucleotides, respectively, and had introns between 50 bp (minimum imposed to avoid gaps in annotation) and 10,000 bp in length. Among the 200,000 unmapped SAGE tags in the Mouse Atlas of Gene Expression Project (detailed below) [25], 261 such tags, which did not map to RefSeq, Ensembl, Mammalian Gene Collection (MGC), or the mouse genome, were found to match these EST-predicted tags. These 261 EST-only tags are distinct from the transcript dataset used in initial parameter selection and junction profile model building, thus providing an independent test set. For each splice junction position within the tags, the percentage of tags correctly mapped by using the optimal *p-*value cutoff values was determined (Figure 6). As illustrated, a minimum length of 5 bp for the shorter edge produces reliable predictions. In many cases, laboratory researchers are prepared to test multiple candidate predictions. Therefore, we investigated, for each length, the number of top-ranking candidates required to detect a true junction (Figure 6). The closer a splice junction is to the center of the tag, the fewer candidates are required to find a validated result. For each tag, by testing the candidate with the lowest *p-*value, investigators can expect 90% of tags to be mapped successfully, if the junction is at least 5 bp from the edge of the tag.

### Unmapped Tag Search Results

We applied SAGE2Splice search to a collection of 20,000 unmapped SAGE tags obtained from the Mouse Atlas of Gene Express Project (http://www.mouseatlas.org). These tags were selected based on tag abundance from a set of LongSAGE libraries. Using default *p-*value cutoffs for donor, acceptor, and composite splice sites, and 10,000-bp maximum intron size, a total of 1,511 tags (7%) were mapped to candidate splice junctions (Dataset S1).

The selection of tags based solely on abundance excludes high-quality tags for rare transcripts. We utilized a second collection of Mouse Atlas of Gene Expression LongSAGE libraries and selected 20,000 high-quality tags based on the method described in Siddiqui et al. [25], a procedure that preserves rare high-quality tags. Of the 20,000 tags, 1,639 (8%) mapped to candidate splice junctions (Dataset S2). Thus, both in silico analyses indicate that a striking portion (7%–8%) of unmapped SAGE tags are consistent with potential splice junctions.

### Candidate Validation

To select candidate junctions for testing, Perl scripts were written to computationally categorize the candidates from the quality-ranked tag collection into tag types. For clarity, the 1,639 tags mapped to 7,757 candidate junctions. We screened all candidate junctions irrespective to edge length. Based on matching both donor and acceptor positions in the UCSC annotation database, 15 candidate junctions were classified as Type 1. There were 803 junctions classified as Type 2, for which either only the donor position or only the acceptor position matched an annotated exon. The remaining 6,939 candidate junctions matched no known exons and were classified as Type 3. By mapping candidates corresponding to Type 2 and Type 3 to exons predicted by GenScan [26], TwinScan [27], or SGP [28], five Type 2 candidates and three Type 3 candidates were categorized as prediction supported. On the basis of RNA sample availability, we picked eight candidates from the Type 1 category, two candidates from the Type 2 category, and two candidates from the Type 3 category for RT-PCR testing (Table 2).

**Table 2:**
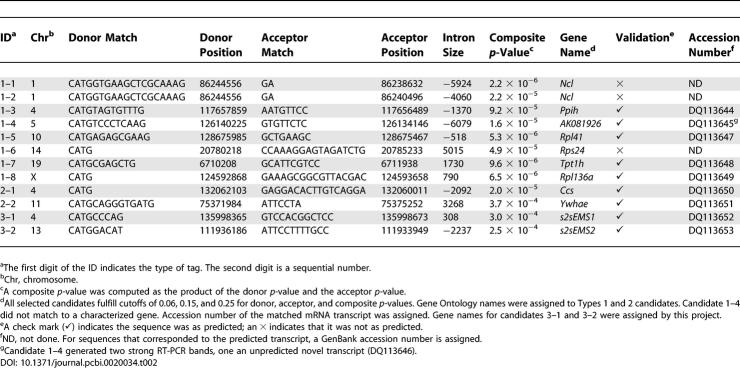
Twelve Candidates Were Selected for RT-PCR Validation

**Figure 7:**
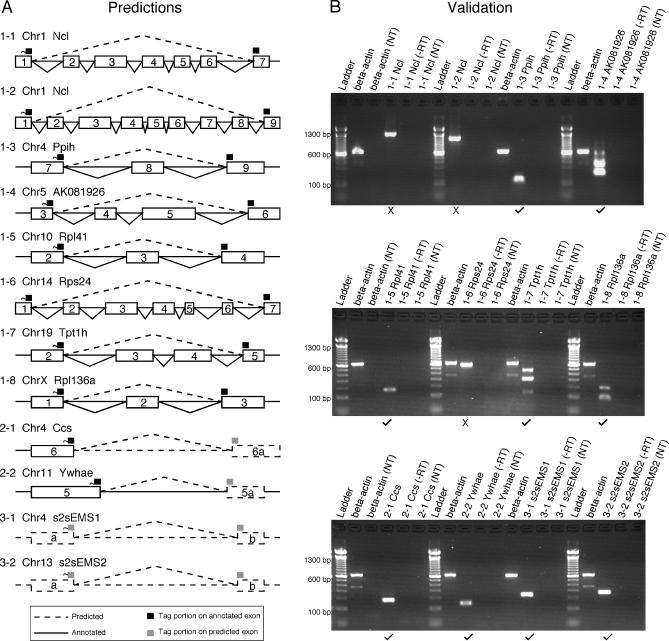
Nine of 12 Selected Candidates Revealed Novel Splice Junctions by RT-PCR and Sequencing (A) Predicted splice junctions of the 12 selected candidates. First digit of the candidate ID indicates the tag type; the second digit is arbitrarily assigned. (B) Except for Candidates 1–1, 1–2, and 1–6, all candidates show the correct product size and were sequence validated. A larger band from an unpredicted novel splice junction was also observed for candidate 1–4. Larger bands were also observed for candidates 1–7 and 1–8, but were shown to be known splice variants. Candidates that were validated by RT-PCR and by sequencing are indicated by a check mark (✓) under the respective lane; candidates not validated, by an ×. NT, negative control with no RNA template; −RT, negative control with no reverse transcriptase.

For the selected candidates, primers were designed based on the contiguous exons predicted by SAGE2Splice (Table 3). RT-PCR results showed that nine of the 12 tested candidates generated products of the predicted length (Figure 7). The other three candidates produced bands that were larger than expected. All of the latter candidate splice junctions were located close to the edges of the SAGE tags. However, two of the nine candidates did have the correct band sizes, even though the candidate splice junctions were located only 4 bp away from the tag edge. Sequencing of the RT-PCR products confirmed the products contained the expected sequences (in addition to matching the expected size). Two strong bands were observed for candidate 1–4, one that matched the size of the expected length (221 bp) and the other one larger (361 bp). Sequence of the expected band corresponded to the novel alternative combination predicted; sequence of the larger product revealed an unpredicted, previously unidentified alternative transcript of the same gene. Unpredicted larger bands were also observed for candidates 1–7 and 1–8 (306 bp and 197 bp, respectively) and corresponded to known transcripts.

**Table 3:**
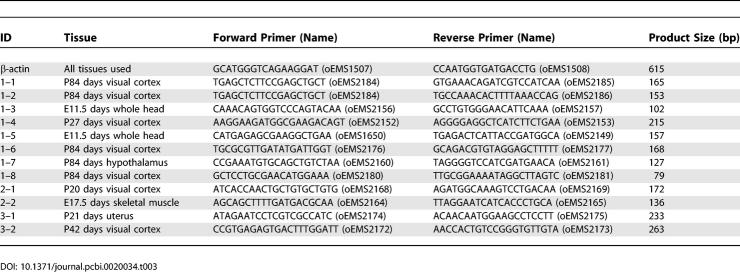
RT-PCR Primers Were Designed for the Selected Candidates Based on Sequences of the Two Predicted Exons

We used sequences from the validated candidates to computationally predict their longest open reading frames (ORFs). Candidates 1–3 and 2–2 encoded short alternative C-terminal sequences (Table 4). Candidates 1–5, 1–7, and 1–8 contained alternative ORFs. Novel ORFs were predicted within candidates 1–4, 2–1, 3–1, and 3–2. Protein–protein BLAST (BLASTP) to the NCBI all-organism non-redundant database showed no significant matches for candidates 1–3, 1–4, 1–5, 1–7, 1–8, and 2–2. Candidate 2–1 matched a dog zinc finger DHHC domain–containing protein. Candidates 3–1 and 3–2 showed significant similarities to previously reported rat proteins: heparin sulfate proteoglycan 2 and integrin alpha 1. Significant matches to known proteins in a different organism are strong evidence that these three predicted transcripts encode functional proteins.

**Table 4:**
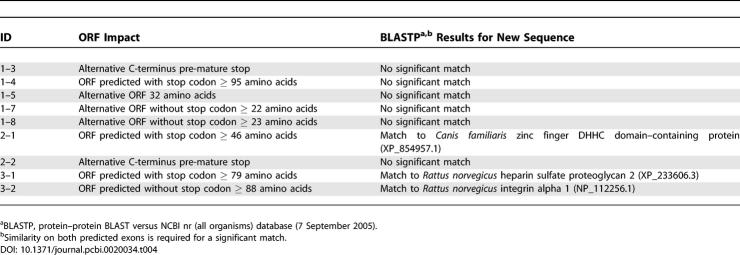
ORF and BLASTP Analyses of the RT-PCR and Sequencing Validated Candidates

### Experimentally Motivated Heuristic Filtering

Of the tested candidates, the two with a shorter edge of 2 bp and one with a shorter edge of 4 bp were not detected by RT-PCR. Conversely, all candidates with a splice junction closer to the center of the tag were confirmed by RT-PCR. These observations are consistent with our Edge Length and Rank Analysis (see above), which suggested a minimum length of 5 bp for reliable predictions. Thus, we recommend eliminating candidates with edges less than 5 bp. Applying this filter to Dataset S1 results in 1,064 tags (5.3%) mapping to 2,588 candidate splice junctions. Applying the filter to Dataset S2 gives 1,212 tags (6.1%) mapping to 3,458 candidate junctions. Together this data predicts that 5%–6% of unmapped tags span a splice junction.

## Discussion

We have developed a tool, SAGE2Splice, for efficient mapping of SAGE tags to potential splice junctions in a genome. By using a scoring system that generates a probability value for each candidate splice junction, SAGE2Splice allows users to assess the quality of the candidates. Furthermore, the in silico validation pipeline automatically classifies the candidates into three categories, based on overlaps with annotated and predicted exons. We identified candidate junctions for 7%–8% of unmapped tags, using parameters designed for high specificity. This is the first attempt to investigate systematically SAGE tags that span splice junctions and to use this characteristic for transcript identification. The online version of SAGE2Splice (http://www.cisreg.ca) allows users to search the genome sequences for human, mouse, rat, and worm, the four most common organisms in NCBI's SAGE database. All source code and data are available for download from the SAGE2Splice Web site.

Scanning a genome for potential splice junctions is computationally challenging. The mouse genome, roughly 3 Gb, takes on the order of several minutes to scan. Disk access dominates the running time when the number of input tags is low. As the number of input tags increases, the search time becomes dominant. Due to the increased probability of observing halftag matches that trigger more computationally intensive searches, longer maximum intron length settings increase runtime. The time efficiency of SAGE2Splice is *O(nm)*, where *n* is the number of input tags and *m* is the size of the genome. Since SAGE2Splice reads and keeps only a fixed length of genomic segment in memory at any time, memory usage is minimal. Memory space is dependent on the number of input tags, and, thus, is defined as *O(n)*, where *n* is the number of input tags.

The portion of tags corresponding to splice junctions in a SAGE library is unknown. Incomplete enzyme digestion or alternative splicing at the 3′ end of a transcript could give rise to multiple tag types from the same gene [13]. Thus, we expect the portion of spliced tags in a SAGE experiment to be higher than 1.6%, which was based on predictions from the 3′-most tags in RefSeq transcripts, but lower than 6.2%, which was based on predicted tags from all positions. Among the high expression and or high sequence-quality unmapped tags, the portion of spliced tags is expected to be higher. In both analyses of unmapped SAGE tags, 7%–8% consistently matched a candidate splice junction when high specificity parameters were used. By applying our recommendation to filter out candidates with a minimum edge length less than 5 bp, this value is reduced to 5%–6% of unmapped tags matching a candidate splice junction. This observation is not inconsistent with the recent recognition of the complexity of the mammalian transcriptome brought about by alternative splicing [1].

One area for improvement of the SAGE2Splice algorithm would be to incorporate methods to also detect non-canonical candidate junctions. As in other studies [23,24], we adopted PWM profiles for splice site detection. In addition, SAGE2Splice uses tag sequence as support and includes a criterion for the presence of the canonical dinucleotide prior to scoring the candidates. This heuristic requirement for the canonical dinucleotide pair limited our searches to about 96.27% of potential splice junctions (according to known splice junctions in RefSeq annotation). We would like to incorporate methods such as decision trees into our splice junction evaluation scheme and, thus, allow SAGE2Splice to detect non-canonical candidate junctions.

SAGE2Splice is demonstrated to be a potent tool for computational prediction of novel splice junctions using unmapped tags. The results indicate that unmapped SAGE tags represent a rich resource for the discovery of novel transcripts. As the annotation of genomes and the characterization of genes and transcripts continue, systematic exploration of candidate novel splice junctions through the use of SAGE2Splice will help elucidate the transcriptome.

## Materials and Methods

### Source of transcripts and known splice junctions.

The genomic sequences of C57BL/6J mouse (mm5, May 2004) and the RefGene annotation database of RefSeq transcripts (July 16, 2004) were obtained from the UCSC Genome Browser [29]. Sequences in RefSeq are considered to be high quality because they have been examined and curated by experts [30]. The UCSC genome annotation pipeline maps the transcript sequences to the mouse genome and identifies the exon coordinates.

For each transcript, the RefGene annotations include the chromosome, the orientation, the exon coordinates, and the translated region coordinates. Based on this information, we developed programming scripts in the Perl language (version 5.6) to re-construct the RefSeq sequences from the mouse genome sequence. These re-constructed RefSeq sequences enabled us to examine the boundary patterns of each splice junction, as well as to analyze the predicted SAGE tags and the number of Type 0 tags.

### Extraction of predicted SAGE tags.

We computationally extracted, from the RefSeq transcript sequences, all predicted SAGE tags, by obtaining 21 bp (LongSAGE) downstream of each NlaIII-anchoring enzyme restriction site. Each predicted tag was annotated with its distance from the 3′ end, which was given the position 0.

### Scoring splice junctions.

For each observed splice junction, we examined the window of 10 bp on either side. By counting the occurrences of each nucleotide at every position, frequency matrices were constructed for donor and for acceptor patterns. Assuming that in a random sequence all four nucleotides have equal probability, we converted these matrices, for every nucleotide at every position, to PWMs [31] by using the formula 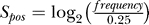 . For each donor and acceptor junction, 10 bp from each side of the boundary were extracted and, by using their respective PWM, a score was computed as 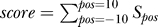 . To generate empirical score distributions for *p-*value assignments, 100,000 sequences of 20 bp in length and containing G and T at the 11th and 12th positions were randomly selected from the genome, and each were scored by the donor PWM. Similarly, 100,000 sequences of 20 bp containing A and G at the ninth and tenth positions, were selected and each scored by the acceptor PWM. Empirical distributions were generated by ranking the scores. For each candidate intron, the proposed donor and acceptor junctions were scored separately, according to their respective matrices. A *p-*value was assigned based on the relative position of the observed score on the junction's empirical distribution. Assuming independence, a composite *p-*value was computed as *p(Donor, Acceptor) = p(Donor)p(Acceptor)*.

### SAGE2Splice implementation and features.

The core program of SAGE2Splice was written in the Perl programming language (version 5.6), and executed by using a compiled version to increase performance. An Internet interface was created by using the PHP scripting language (http://www.php.net). In addition to providing a list of SAGE tags as inputs, the user has the options of specifying the following: the anchoring enzyme recognition sequence (default is NlaIII, CATG), the maximum intron size (default is 10,000 bp), and the cut-off *p-*values for the donor candidate, the acceptor candidate, and the composite candidate (defaults are 0.06, 0.15, and 0.0025, respectively). The implementation of SAGE2Splice allows the user to adapt to different organisms simply by modifying the configuration file. The SAGE2Splice program and the Web interface PHP script, are available for download (http://www.cisreg.ca).

### Efficiency tuning of SAGE2Splice.

We tested a series of different genomic-segment size settings to find an optimal size for computational efficiency. Tested sizes include: 10, 20, 30, 40, 50, 60, 70, 80, 90, 100, 200, 300, 400, 500, 600, 700, 800, 900, and 1,000 kilobasepairs. For each size, we performed five iterations of the SAGE2Splice algorithm to search for ten randomly selected SAGE tags, and recorded the average execution time in seconds. Efficiency analysis was performed on a 14-node cluster, in which each node had two Intel Xeon processors at 2.4 GHz with 1.5 GB random access memory running Red Hat Linux version 7.3 (Red Hat, Raleigh, North Carolina, United States). Perl version 5.6 (O'Reilly Media, Sebastopol, California, United States) was used to compile the core SAGE2Splice program.

### Sensitivity and specificity.

We randomly chose from the list of predicted tags 1,000 tags that were known to span a splice junction to have GT and AG as the junction dinucleotide pairs, and to have the introns within 10,000 bp of each other, as our positive controls for testing SAGE2Splice. By searching against the corresponding genome using SAGE2Splice, true positives (TP) were identified if the original splice junctions were found, and false negatives (FN) were identified if no known splice junction was found. For negative controls, we chose from the same predicted tag lists, 1,000 tags that were known not to traverse a splice junction. A true negative (TN) evaluation is when no candidate was output by SAGE2Splice, whereas a false positive (FP) identifies a candidate junction for a negative tag. Sensitivity of SAGE2Splice was computed as 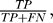 whereas specificity was computed as 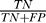 .

### Source of SAGE tags.

In searching for novel transcripts, we utilized the SAGE data generated from the Mouse Atlas of Gene Expression Project [25]. The Atlas project aims to examine comprehensively and quantitatively the expression of genes of various organ and tissue types throughout the development of mouse, from a single-cell zygote to the adult. For genetic homogeneity, throughout the project only the C57BL/6J strain of mouse was used for library construction. At the end of the project, 200 SAGE libraries will have been generated. The LongSAGE protocol [20], which is similar to the original SAGE [5] in preparation, but generates 21-bp tags, is being used in the majority of the SAGE libraries constructed. In this study, only the 21-bp tags were used. All SAGE data and analysis tools are public and can be downloaded from the Web (http://www.mouseatlas.org).

### Searching unmapped SAGE tags.

In the Mouse Atlas of Gene Expression Project [25], SAGE libraries that were completed and in progress of construction during the period of January 2005 to September 2005 were pooled to generate a meta-library. The abundance of each tag type is summed. We exhaustively mapped the tags in this meta-library to all predicted tags extracted from RefSeq [30], Ensembl transcripts, MGC [32], mRNA sequences, EST collections, and the C57BL/6J mouse genome (NCBI Build 33), as well as to the full mouse UniGene mapping of SAGEmap (Build 145) [15], and then we selected the 20,000 most abundant SAGE tags for SAGE2Splice searches against the C57BL/6J mouse genome sequence (NCBI Build 33). We used the default 10,000-bp maximum intron length and *p-*value cutoffs of 0.06, 0.15, and 0.0025 for the donor, the acceptor, and the overall score, respectively.

To include in the search those rare tags that were of high quality, we pooled a separate meta-library based on the libraries completed or in progress before January 2005. As described by Siddiqui et al. [25], each tag sequence was assigned a quality factor, which was computed by using PHRED scores [33], and a tag sequence probability value (*p-*value) was assigned based on the quality factor and the rate of errors in library construction. For tags observed more than once, individual *p-*values were multiplied to obtain a composite *p-*value. The more frequent the observations, the more confidence in the existence of the tag, thus resulting in a lower *p-*value. The tags in this meta-library were ranked by their composite *p-*values, and SAGE2Splice search was applied to 20,000 tags with the lowest *p-*values using the same criteria as the previous dataset.

### Categorization of splice junction candidates.

Three pipelines were created to classify the candidates into their respective categories. We obtained, from the UCSC Genome Browser, transcript annotations, including RefSeq, Ensembl transcripts, MGC, mRNA sequences, and EST collections, and gene predictions annotations, including TwinScan [27], GenScan [26], and SGP Gene [28]. Candidates returned by SAGE2Splice were categorized by matching candidate junction positions to those in known transcripts. Candidates associated with Type 2 and Type 3 tags were further categorized by mapping the candidate junction positions to gene prediction annotations. Candidates that mapped to predicted junctions were classified as high priority in the validation list.

### RNA extraction.

All samples were manually dissected and stored at −80 °C until RNA extraction. Frozen tissue was disrupted and homogenized for 30 s with a Polytron PT 1200CL homogenizer (Kinematica, through Brinkmann Instruments, Mississauga, Canada) at a setting of 3 (~13,000 RPM), equipped with a 7-mm generator (PT-DA 1207/2EC). RNA from each sample was extracted by using either RNeasy Mini Kit or RNeasy Lipid Tissue Mini Kit (Qiagen, Mississauga, Canada), with an on-column DNaseI treatment. Quality assessment and quantification of each RNA sample was done by using RNA 6000 Nano LabChip Kit on an Agilent 2100 Bioanalyzer (Agilent Technologies Canada, Mississauga, Canada). Tissue samples of embryonic (E) 11.5 whole head (rEMS315, from EMS laboratory collection), post-natal day (P) 84 hypothalamus (rEMS340), P21 uterus (rEMS341.01), and E17.5 skeletal muscle (rEMS344) were processed by using the RNeasy Mini Kit protocol. Samples of visual cortex P20 (rEMS300), P27 (rEMS301), P42 (rEMS304), and P84 (rEMS305) were processed by using the RNeasy Lipid Tissue Mini Kit following manufacturer's directions with the modification of using 1.5 ml Phase Lock Gel-Heavy tube (Eppendorf Scientific, through Fisher Scientific, Ottawa, Canada) for more robust phase separation. All tissues were extracted from male C57BL/6J mice, except for the uterine tissue (rEMS341).

### RT-PCR.

Primers for each candidate (Table 3) were designed by using Web Primers provided by the Saccharomyces Genome Database (http://www.yeastgenome.org). RT-PCR amplification was performed with the QIAGEN OneStep RT-PCR Kit (Qiagen) as per the manufacturer instructions. Reverse transcription was performed at 50 °C for 30 min. Amplification reactions included 0.4 mM of each dNTP, 1× QIAGEN OneStep RT-PCR buffer, 1× Q-Solution 2.0 μl QIAGEN OneStep RT-PCR Enzyme Mix per 50 μl reaction, and 5 U RNase inhibitor (Invitrogen Canada, Burlington, Canada) per reaction. Reverse transcriptase inactivation and PCR activation were performed at 95 °C for 15 min, followed by 40 cycles of 94 °C for 30 s, 58 °C for 30 s, and 72 °C for 1 min, and a final extension step at 72 °C for 10 min. Candidates 1–3, 1–5, and 1–8 were performed at 55 °C, 30 s for annealing. For the negative controls with no reverse transcriptase, the RNA was not added until after the reverse transcriptase inactivation step.

## Supporting Information

Dataset S1 Results of SAGE2Splice Search of a Collection of 20,000 High-Abundance SAGE Tags The prediction splice junctions of each search are further categorized into Type 1, Type 2 with computer prediction, Type 2 without computer prediction, Type 3 with computer prediction, and Type 3 without computer prediction. A detailed description is available in the README file.
(882 KB ZIP)

Dataset S2 Results of SAGE2Splice Search of a Collection of 20,000 High-Quality SAGE Tags The prediction splice junctions of each search are further categorized into Type 1, Type 2 with computer prediction, Type 2 without computer prediction, Type 3 with computer prediction, and Type 3 without computer prediction. A detailed description is available in the README file.
(817 KB ZIP)

### Accession Numbers

The National Center for Biotechnology Information (NCBI) (http://www.ncbi.nlm.nih.gov) accession numbers for the sequences discussed in this paper are *Canis familiaris* zinc finger DHHC domain–containing protein (XP_854957.1), Rattus norvegicus heparin sulfate proteoglycan 2 (XP_233606.3), and R. norvegicus integrin alpha 1 (NP_112256.1). The GenBank (http://www.ncbi.nlm.nih.gov/Genbank) accession numbers for the sequences discussed in this paper are *AK081926* (DQ113645),*^,^ Ccs* (DQ113650), *Ppih* (DQ113644), *Rpl136a* (DQ113649), *Rpl41* (DQ113647), *s2sEMS1* (DQ113652), *s2sEMS2^,^* (DQ113653), *Tpt1h* (DQ113648), and *Ywhae* (DQ113651).

## Abbreviations

bp: basepair
EST: expressed sequence tag
MGC: Mammalian Gene Collection
NCBI: National Center for Biotechnology Information
ORF: open reading frame
PWM: position weight matrix
ROC: receiver operating characteristic
SAGE: serial analysis of gene expression
UCSC: University of California Santa Cruz
